# The Next Frontier in Communication and the ECLIPPSE Study: Bridging the Linguistic Divide in Secure Messaging

**DOI:** 10.1155/2017/1348242

**Published:** 2017-02-07

**Authors:** Dean Schillinger, Danielle McNamara, Scott Crossley, Courtney Lyles, Howard H. Moffet, Urmimala Sarkar, Nicholas Duran, Jill Allen, Jennifer Liu, Danielle Oryn, Neda Ratanawongsa, Andrew J. Karter

**Affiliations:** ^1^University of California, San Francisco, CA, USA; ^2^Arizona State University, Tempe, AZ, USA; ^3^Georgia State University, Atlanta, GA, USA; ^4^Kaiser Permanente, Oakland, CA, USA; ^5^Redwood Community Health Coalition, Petaluma, CA, USA

## Abstract

Health systems are heavily promoting patient portals. However, limited health literacy (HL) can restrict online communication via secure messaging (SM) because patients' literacy skills must be sufficient to convey and comprehend content while clinicians must encourage and elicit communication from patients and match patients' literacy level. This paper describes the Employing Computational Linguistics to Improve Patient-Provider Secure Email (ECLIPPSE) study, an interdisciplinary effort bringing together scientists in communication, computational linguistics, and health services to employ computational linguistic methods to (1) create a novel Linguistic Complexity Profile (LCP) to characterize communications of patients and clinicians and demonstrate its validity and (2) examine whether providers accommodate communication needs of patients with limited HL by tailoring their SM responses. We will study >5 million SMs generated by >150,000 ethnically diverse type 2 diabetes patients and >9000 clinicians from two settings: an integrated delivery system and a public (safety net) system. Finally, we will then create an LCP-based automated aid that delivers real-time feedback to clinicians to reduce the linguistic complexity of their SMs. This research will support health systems' journeys to become health literate healthcare organizations and reduce HL-related disparities in diabetes care.

## 1. Introduction

“The single biggest problem in communication is the illusion that it has taken place.” George Bernard Shaw.

Health literacy (HL) refers to a patient's capacity to obtain, process, communicate, and understand basic health information and services needed to make appropriate health decisions [1]. Limited HL can place individuals at greater risk of type 2 diabetes (DM2) and its complications, making limited HL a critical clinical and public health problem [2, 3]. Suboptimal communication exchange, and resultant problems with* medication* adherence, is a mediator between limited HL and DM2 outcomes. However, effective communication between patients and providers can mitigate the impact of limited HL. Online patient portals that support asynchronous, between-visit electronic communications (secure messages [SM]) are heavily promoted by health systems and patient uptake is high [4, 5]. Secure messaging (SM) represents a “disruptive” innovation that is rapidly expanding (~5–10%/year) in systems such as Kaiser Permanente (KP), thereby complementing, and at times, supplanting, or stimulating in person/visit-based communication. Enabling patients with DM2 to easily access their medical information is a novel approach to facilitate patient engagement and activation; empowering patients in this fashion improves disease knowledge, enhances patient-provider communication, and increases adherence to treatment [6]. While research demonstrates that patients who access portals are more likely to have favorable healthcare utilization patterns, adhere to prescribed regimens, and achieve better outcomes, minority status, low levels of income, limited HL, and older age are all associated with lower portal usage. In addition, patients specifically require a certain degree of linguistic facility to take advantage of SM as a means of communication, and patients with limited HL may have difficulty messaging their provider or understanding the provider's replies or instructions. Providers too must engage with patients in a manner that provides* meaningful and actionable information *and support in an easily comprehended style that promotes shared meaning.

Secure messaging has been shown to be particularly relevant for patients with DM2, given their need for relatively frequent communication between outpatient encounters. No studies have examined how DM2 patients with limited HL and their providers interact via SM. In the Employing Computational Linguistics to Improve Patient-Provider Secure Email (ECLIPPSE) study, funded by National Library of Medicine, we examine to what extent clinicians accommodate the communication needs of their DM2 patients with limited HL. The IOM recently advocated that health systems and clinicians must accommodate the communication needs of patients with limited HL to overcome the challenges facing this clinical vulnerable population. The degree to which providers adjust linguistic complexity in their SM exchanges to “match” the level observed in their patients will serve as one indicator of the extent to which providers are, or are not, making such accommodations.

**Figure 1:**
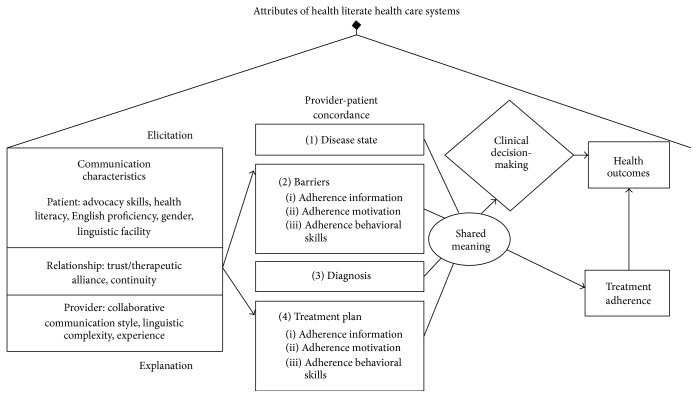
Conceptual Framework for concordant health communication in DM2 care in the Patient-Centered Medical Home.

This study integrates 3 conceptual frameworks and theories to assess the effect of LCP discordance in SM on a range of DM2 outcomes.* The Conceptual Framework* (Figure 1) integrates 3 complementary research and operational paradigms: (1) communication in Chronic Illness Care Framework of Schillinger [7], (2) Information-Motivation-Behavior Model (IMB) of DM2 medication adherence [2], and (3) IOM attributes of “health literate healthcare organizations.” [8] Our framework revolves around achieving provider-patient concordance and shared meaning across 4 domains involving* elicitation-type communication* and* explanatory-type communication*. Communication concordance can improve chronic disease outcomes [9, 10]. We will examine whether patient LCP is associated with medication adherence, HbA1c, hypoglycemia, or long-term outcomes. We augmented this framework with the IMB Model of DM2 medication adherence [11]. IMB elements explain >40% of variance in adherence and predict glycemic control, even in low SES populations [2]. Interventions that target IMB have been successful in previous studies [12]. In IMB, adherence is determined by the extent individuals are* informed* about their regimen,* motivated* to adhere, and possess or are provided with* skills*. All 3 domains of IMB are sensitive to interactive patient-provider communication, making them appropriate targets for discussions of barriers to adherence (Figure 1, Box 2) or treatment (Figure 1, Box 4). In 2012, the Institute of Medicine commissioned a White Paper to define attributes of “health literate healthcare organizations” [8]. This paper served as a national call to shift focus from HL skills of individuals toward HL-promoting actions of organizations, including providers. Five of 10 attributes bear relevance to this proposal: (1) meeting needs of populations with a range of HL; (2) using HL strategies in interpersonal communications and confirming understanding; (3) providing easy access to health information/services; (4) designing and distributing content that is easy to understand and act on; and (5) preparing workforce to be health literate [8]. Many providers are unprepared for communicating with patients with limited HL [4] and lack tools to improve communication-sensitive outcomes. A goal of this proposal is to create an automated communication aid prototype, based on provider LCPs, that delivers feedback to providers to reduce linguistic complexity of SMs. Implementation of this tool could advance an organizations' journey to become more health literate.

## 2. Materials and Methods

Our study involves 2 settings. The first,* Kaiser Permanente Northern California (KPNC)*, is a nonprofit, fully integrated healthcare delivery system, providing services through 37 outpatient centers and ~3,300 providers to 3.3 million plan members, in a 14-county region of Northern California. Except for extremes of income, sociodemographic characteristics of KP members are representative of the local population [13]. KP provides care to a population insured through employer-based plans, Medicare, Medicaid, and new health insurance exchanges. Thus, our study findings should be widely generalizable outside of KP. KP has a very well-developed, mature patient portal, kp.org, used by KP members to SM their healthcare team. KP maintains integrated administrative and clinical databases (pharmacy, lab, diagnoses and procedures, clinical notes, SMs, utilization, and cost) linked to individual members. Part of our sample will be drawn from the KP DM2 Registry (*n* = 229,027 between 1/06 and 6/14). Descriptions of the Registry have been published previously [14–17]. Nested within KP DM2 Registry are ~11,000 DM2 patients from the well-characterized DISTANCE cohort [18], for whom we have measures of self-reported HL [19], patient reports of provider's communication quality, and a broad array of sociobehavioral and psychological measures obtained in 2006 [20]. Nearly 40% of DISTANCE respondents had limited HL, and the cohort is diverse (19% Hispanic, 17% African American, 23% Asian/Pacific Islander, 23% White/non-Hispanic, and 18% Multiracial). To achieve some of the aims of ECLIPPSE, we will use data from KP's patient portal, which includes SM capabilities. The second site,* The San Francisco Health Network (SFHN)*, is a public delivery system that includes the following: 13 primary care centers and specialty and inpatient services at SF General Hospital and provides >1.5 million visits and 24-hour care to ~120,000 low income patients annually. SFHN patients are insured by Medicaid (39%), Medicare (28%), and commercial health insurance (2%) or are of no insurance (32%, a number falling due to ACA). The population is 30% Hispanic, 20% African American, 32% Asian/Pacific Islander, 13% White/non-Hispanic, 3% Native American, and 2% Multiracial. SFHN cares for 8,105 DM2 patients who have seen their primary provider (*n* = 270) >once in the prior year; ~50% have limited HL [21–26]. In 2013, 42% of SFHN DM2 patients had HbA1c >8% (versus 32% in KP). The SF Department of Public Health created The Hospital Record Electronic Data Set (THREDS) that contains demographics, pharmacy, lab, diagnoses, and utilization. In 10/14, SFHN's EHR added a self-reported HL measure: “how confident are you filling out medical forms by yourself?” [27]. SFHN launched* mySFHealth* patient portal [28] in 10/14, which includes SM functionality in 2015. We anticipate that >10% of DM2 patients from SFHN will engage in SM by 2016, 20% in 2017, 30% by 2018, and 35% by 2019 (*n* = 2,837,225 providers) [29].

Our study includes 3 aims.* The first aim* is to develop and validate a novel, automated Linguistic Complexity Profile (LCP) to assess secure message content generated by English-speaking DM2 patients and their providers. We will employ natural language processing (NLP) indices to develop and validate the LCP, based on >5 million SM and covariate data from >150,000 ethnically diverse, DM2 patients receiving care in 2 integrated health systems: a large, integrated, healthcare delivery system with a mature patient portal (Kaiser Permanente Northern California, KPNC) and a county-run, integrated, public (safety net) delivery system with a newly launched patient portal (San Francisco Hospital Network, SFHN). We will first aggregate our selected automated linguistic indices into larger components using a principal component analysis [30, 31]. A PCA examines cooccurrence patterns among the selected linguistic indices and, using these cooccurrence patterns, we will develop larger component scores related to linguistic complexity. We will use these component scores in machine learning algorithms to patients' self-reported HL when applied to SMs from ~12,000 patient SMs. We will also use the component scores to predict 9,535 DM2 patients' ratings of physicians' communication from 950 providers. These analyses will provide construct validity for the component scores and be used as the foundation of the LCP. We will then use the LCP models derived from the patient's self-reported HL and ratings of physicians' communications to determine, among >150,000 DM2 patients, whether patient LCP is associated with HbA1c, adherence for cardiometabolic medications, DM2 complications, hypoglycemia, and utilization of services.

We hypothesize that (1) patients' and their providers' LCP will demonstrate construct validity based on patients' HL level and reports of provider communication (using the AHRQ Consumer Assessment of Healthcare Providers & Systems score, CAHPS), respectively; and (2) patient LCP will correlate with clinically relevant outcomes, for example, med adherence, HbA1c, hypoglycemia, and utilization of services. Patients' HL level will be based on a previously validated instrument [19, 32] This item has test characteristics similar to the cumulative 3-item scale and is validated against in person, interview-based tools, such as REALM and TOFHLA (areas under ROC: 0.70–0,82) [19, 27]. We have shown its predictive value for DM2 outcomes in DISTANCE [27, 33]. We will use the CAHPS instrument to assess provider communication skills. Provider LCP will be assessed for construct validity against items from the patient-reported, provider communication subscale (over 12 months, how often their provider* listens carefully* to them;* explains things* in a way they could understand;* spends enough time* with them; and* involves them* in making decisions about care) from the CAHPS survey [34] in DISTANCE. We will calculate a summary CAHPS score [35] (range, 0–100) by linearly transforming and averaging responses (Cronbach *α* in DISTANCE = 0.80) [20]. We will only measure provider LCP during the period the patient reported CAHPS. We hypothesize that higher CAHPS will correlate negatively with LCP of providers' SMs. CAHPS data were captured by a previous survey conducted by the Diabetes Study of Northern California (DISTANCE) [18].

*The second aim* is to examine whether concordance between providers' and patient's LCP is associated with better adherence among DM2 patients newly prescribed insulin or an antidepressant, excluding patients with pharmacy dispensing in prior 2 years. We have previously shown that limited HL predicts poor adherence to communication-sensitive medications, for example,* insulin* and* antidepressants *in DM2 [33, 36]. Based on prior analyses, we estimate ~52,000 patients in KP Registry will have started insulin and ~28,000 antidepressants (selective serotonin reuptake inhibitor or serotonin norepinephrine reuptake inhibitor, mirtazapine or bupropion) during our study period [33]. We hypothesize that greater degrees of patient-provider concordance LCP in the period surrounding the start of insulin or antidepressant will be associated with better adherence to these newly prescribed medications. Our outcome measure will combine primary nonadherence (never filling Rx) and early nonpersistence (never refilling Rx). These standard adherence measures are obtained via pharmacy claims 6 months after initiation [20, 33, 37].* The third aim* is to create an automated, LCP-based prototype to deliver automated feedback to providers to reduce their SM linguistic complexity and enhance shared meaning. After developing the prototype, we will enlist 42 providers to evaluate whether the prototype improves LCP concordance using a series of in vitro experiments with simulated, standardized clinical scenarios. We evaluate whether it improves LCP concordance for DM2 providers via 3-arm randomized controlled trial (RCT) design. Formative feedback must be timely, impersonal, suggestive, digestible, and not interrupting workflow [38]. Providers will be given automated feedback on SMs as they draft them. Issues flagged will be related to the SM (not the individual provider) and to potential (not definitive) problems. Feedback content will be developed and piloted with the team's primary care physicians to ensure it is impersonal, suggestive, digestible, and actionable.* Arm 1 (active control feedback)* will receive automated linguistic feedback based on NLP algorithm related to socioemotional tone, rapport building, and degree of empathy and support. Support was selected as an active control because (a) it has face validity with providers, (b) we expect a high proportion to receive feedback [39], and (c) there is no evidence that increasing socioemotional content of SMs affects linguistic complexity [40, 41].* Arm 2 (Flesch-Kincaid feedback)* will receive automated linguistic feedback based on Flesch-Kincaid's algorithm [42], selected as a comparison because (a) it is ubiquitous and recognizable, (b) it generates grade level of text, which has face validity with providers, and (c) our pilot work suggests providers rarely generate SMs at <6th grade level; therefore, we expect a high proportion will receive feedback. In* Arm 3 (LCP-based feedback)*, the LCP developed in Aims 1 and 2 will provide algorithms that assess complexity of patient and provider SMs. Algorithms will be translated to linguistic feedback to guide providers to generate more concordant SMs. The nature of feedback will allow for more granular, specific, and tailored feedback than Flesch-Kincaid, which we hypothesize will more likely lead to linguistically concordant SMs. We hypothesize that this automated communication aid deployed in clinical simulations can reduce provider's linguistic complexity to better accommodate DM2 patients' linguistic skills and HL.

## 3. Preliminary Results

Our preliminary research suggests that patients who access portals, albeit not necessary using SM, are more likely to have better (a) healthcare utilization [43], (b) prescription adherence [37], and (c) glycemic control [44, 45]. Among DM2 patients, better ratings of physician communication are associated with greater SM usage [46]. While we found that limited HL posed a barrier to portal and SM use [29], disparities have rapidly narrowed. In 2014, 68% versus 84% of Kaiser Permanente DM2 patients with limited versus adequate HL, respectively, accessed the portal. Overall, 46% used SM in 2014, compared to 30% in 2009. Those with limited HL are rapidly gaining ground, with a 65% increase in 5 years, compared to a 41% increase for adequate HL (20%–>33% for low HL versus 39%–>55%, with greatest gains among Latinos and African Americans) (unpublished data, 2014).

Our research further shows that SMs serve as a critical mode of communication of clinically relevant matter in DM2. Among a sample of DM2 subjects (*n* = 9,535), a mean of 19 SMs involving a mean of 8 SM threads (closed communication loops) were generated annually. While prevalence of SM use in this sample increased 53% (30 to 46%) from 2009 to 2014, the number of outpatient visits in SM users went* down* 4% (from 13.2 to 12.7 total visits per year) during the same timeframe. A SFHN study just before launch of their portal revealed 60% of safety net patients used email, 71% were interested in SM, and 19% currently use email with providers [47]. For SFHN, pilot work with 22 patients who engaged in email with providers in 2013 revealed a mean of 5 SMs, yielding a projection of ~15,000 SMs by 2019, assuming 35% uptake of SM among DM2 patients [48]. The KPMC DM2 Registry cohort who sent ≥1 SM to DM2 providers from 2006 to 2014 (*n* = 151,804) generated >1.5 million SMs in 2013 alone. We also calculated the number of patients from the KPNC Registry who initiated insulin or antidepressants from 2006 to 2010 and the proportion with SMs 3 months before and 3 months after initiation. We found that 37,628 patients (~25%) had insulin initiated in this timeframe: 7,264 (~20%) had SMs in the 3 months before, 9,231 (~25%) after, and 5,720 (~15%) before and after. For antidepressants, 20,440 DM2 patients (~13%) initiated in that timeframe. Of these, 16% had SM exchanges 3 months before, 20% after, and ~12% both before and after.

To further understand SM communication, we conducted a pilot examination of SM content among 50 ethnically diverse DISTANCE respondents. Major themes included the following: provision/explanation of lab/diagnostic tests; requests for/discussion of medications and side effects; requests for follow-up appointments and discussions about specialty visits; reports of symptoms/self-care; and preventive care and DM2 guideline-related reminders. In parallel, we carried out a study of email exchanges between 22 SFHN patients and their primary care physicians in 2013. The most common patient requests were the following: action regarding medication or treatment; lab tests, x-rays, and other studies; referral requests; information regarding symptoms, tests, or procedures; and information regarding medications, side effects, or treatments. Patients requested action in 77% of threads. The most common requests were for a prescription (22%), appointment (21%), clarification (16%), medical guidance (14%), and paperwork (13%), resulting in 62 physician actions. We found that SM content was highly relevant to DM2 patients' clinical care in both health systems [48]. We also identified high degrees of clinical jargon use in clinicians' SMs [49].

Qualitative analyses such as those reported above are time and resource intensive. To address these issues, we have shown that NLP tools involving automatic extraction of linguistic features using computer programming can supplant human ratings to a degree. NLP provides information about language at multiple levels and dimensions [50, 51] and affords the ability to glean just about any aspect of text, language, or discourse. Many NLP techniques are specialized, providing information about different aspects of language. A distinctive aspect of this study is that we will incorporate indices from a* variety* of tools to paint a rich picture of language, discourse, and communication patterns. We will also develop new NLP techniques for our data. We will use Coh-Metrix [50, 52], NLP tool developed by our team, which integrates various NLP indices, including pattern classifiers, part-of-speech taggers [53], syntactic parsers [54], and Latent Semantic Analysis [55]. This is the most commonly used tool to measure linguistic complexity on a broad profile of language [41, 56]. Other NLP tools developed by our team will augment our analyses. The Writing Assessment Tool (WAT) [57, 58] provides computational indices related to* writing quality:* global cohesion, contextual cohesion, rhetorical strategies, and *n*-gram (contiguous sequence of *n* items from a sequence of text or speech) accuracy. The Tool for the Automatic Analysis of Lexical Sophistication (TAALES) [59] examines text features specific to* sophistication of word use*: lexical frequency and range, academic words, concreteness, and meaningfulness. The Tool for Automatic Assessment of Cohesion (TAACO) [60] provides additional indices related to* text cohesion* including word, lemma (mental abstraction of a word to be uttered or written), argument, and synonym overlap between sentences and paragraphs. The Sentiment Analysis and Cognition Engine (SEANCE) provides an overview of a text's affective, cognitive, and social features.

We have used NLP tools to assess linguistic complexity to estimate text comprehensibility.* Cohesion*, a construct central to Coh-Metrix and TAACO, is the degree that relations between concepts are explicit in text by using cues such as word overlap and connectives. High cohesion text enhances reading comprehension, particularly for less skilled readers with less knowledge. Coh-Metrix indices related to text cohesion, lexical sophistication, and syntactic complexity have been used to develop readability measures [50, 61–63]. In contrast, a popular index of readability, Flesch-Kincaid's formula, estimates ease of readability of text by deriving ratios among only 3 linguistic units: numbers of syllables, words, and sentences. NLP indices found in tools such as Coh-Metrix demonstrate significant improvements in predicting readability compared to indices such as Flesch-Kincaid [64] and provide better theoretical overlap with cognitive models of text processing and comprehension [62]. Previous studies have reported that Coh-Metrix indices also perform better than traditional readability formulas in distinguishing among texts simplified to beginning, intermediate, and advanced reading levels [63]. Coh-Metrix, WAT, TAALES, and TAACO also predict quality of writing using linguistic indices related to lexical sophistication, syntactic complexity, and cohesion [57–59, 63, 65–67]. Measures of linguistic complexity and individual differences can also be used to examine links between writing skills and reading comprehension [68]. We have investigated relationships between latent factors underlying writing development and found correlations as high as *r* = .54 between writing quality and reading comprehension [69]. Collectively, these studies demonstrate that linguistic features can be used to examine text complexity, readability, comprehensibility, and links between reading comprehension and writing skills.

In a pilot study, we assessed feasibility of using automated NLP queries to examine SM content in a stratified random sample of DM2 subjects. We were able to efficiently and reliably capture and distinguish provider SMs, patient SMs, and system-generated messages. These preliminary analyses used indices from TAALES and TAACO to examine the potential of developing LCPs from SMs. We examined 402 SMs generated by 13 providers and 51 English-speaking DM2 patients, stratified into 25 low and 26 high HL. We were able to assess provider-patient communication differences in SMs using a number of linguistic features. Preliminary findings were promising. Indices related to word frequency and entropy (i.e., number of documents or contexts in which a word occurs) distinguished SMs by low versus high HL patients, with high HL patients using more infrequent (less familiar) and more specific words that occur in fewer contexts (*p* < .05). Providers used more complex linguistic features than patients, producing more rare words (e.g., jargon), specific words, words with fewer associations to other concepts, and less semantic overlap between paragraphs (*p* < .05). Providers judged by patients to be less communicative on CAHPS used more infrequent (unfamiliar) words and word sequences. This preliminary work suggests that (a) providers' use of complex linguistic features better correlated to high than low HL patients (i.e., providers and low HL patients demonstrated discordance in language use, *p* < .05) and (b) providers modify linguistic output based on patient HL, using more familiar words with low HL patients than high HL patients (*p* < .05), although the gap between providers and adequate HL patients remained large. The findings suggested the feasibility of developing patient LCPs to assess construct and predictive validity with respect to DM2 outcomes, analyzing complexity of providers' SMs, and using LCP to provide feedback to providers.

## 4. Discussion

Most clinicians are untrained in communicating with DM2 patients with limited HL, and health systems have no feasible means to identify patients with limited HL or those clinicians who need support in communicating effectively [4]. The Linguistic Complexity Profile (LCP) presented in this project represents methodological and measurement research focused on HL of individual providers and health systems that can facilitate comparisons across ethnicity and health system settings. Studying whether LCP gaps between provider and patient influence medication adherence reflects basic research into how HL impacts health processes and outcomes. Our communication aid based on LCP represents applied research addressing issues pertinent to HL.

Limited HL places individuals at risk of DM2 and its complications, making it a critical public health problem [2, 3]. In many settings, limited HL, as well as numeracy, has been associated with higher prevalence of DM2, poor glycemic control [21, 70], DM2 complications [21, 71], hypoglycemia [29], and poorer medication adherence [33, 72]. Inadequate health communication is a mediator of the relationship between limited HL and DM2 outcomes [7]. Provider communication shortcomings limit the effectiveness of DM2 self-management interventions, especially for patients with limited HL [9, 23]. DM2 providers often are poorly prepared to communicate with patients with limited HL [4, 5]. Secure messaging (SM) represents a “disruptive” innovation that is rapidly expanding (~5–10%/year) in systems such as KPNC, thereby complementing, and at times, supplanting, or stimulating [73–75] in person/visit-based communication. SM is especially important for patients with DM2, who use SM for critical self-management functions, use portals more often than healthier patients [76], and often have competing priorities during in person visits, creating greater communication needs between visits [75]. CMS has promoted meaningful use of electronic health records (EHRs) via substantial incentives (>$25 billion to date) for clinicians and systems that implement EHRs, encouraging patients to engage in EHR [77]. To receive incentives, systems must demonstrate that significant proportions of patients register for the EHR and exchange SMs with providers. While this program has stimulated uptake of SM in the private sector and has established SM as a new standard of care in the US, SM is now enjoying uptake in safety net and public healthcare systems which disproportionately care for patients with limited HL. However, there is very limited understanding of the impact of SM use across HL levels. Additionally we are unaware of intervention work to enhance the effectiveness of clinician SM use with DM2 patients with limited HL. We do know that ratings of physicians' communication are associated with DM2 medication adherence [20] and that medication adherence is an important target for health communications and HL research. Poor adherence in DM2 is common and associated with higher costs and worse outcomes [2]. Limited HL predicts poor adherence to communication-sensitive medications, for example,* insulin* and* antidepressants *in DM2 [33, 36, 78]. To take full advantage of patient portals, patients must have the linguistic competencies to* convey* and* comprehend* clinical content from providers. Providers' must encourage and elicit patient communication and must match or approximate patients' linguistic complexity levels to enhance SM effectiveness (“linguistic complexity concordance”). These problems underlie the need to develop and test the LCP described in this paper. The ECLIPPSE Study will be the first systematic study of SM between DM2 patients and their clinicians and, to our knowledge, is the first study to employ computational linguistics to analyze and improve digital patient-provider communications.

## 5. Conclusions

This research has the potential to achieve important gains in the effort to translate both diabetes and HL research into meaningful action. First, measuring individual HL in healthcare populations is widely recognized to be a daunting and time-consuming undertaking, making it infeasible and cost-prohibitive to carry out. If the patient LCP proves to be a valid indicator of individual HL and is predictive of health outcomes in DM2, then health systems will have an efficient, automatic method to harness “big data” in order to identify patients with limited HL and that may benefit from outreach and additional self-management support. In addition, if patient-clinician discordance in LCP is found to be prevalent and is associated with suboptimal communication-sensitive outcomes, such as medication nonadherence or hypoglycemia, then health systems can target communication training efforts for those clinicians. Finally, if ECLIPPSE's LCP-based prototype communication aids to provide real-time feedback to clinicians is found to be both feasible for clinicians and effective in increasing the degree of linguistic matching, then clinicians will be much better equipped to promote shared meaning in their communications with vulnerable patients. Insofar as electronic patient portals are being heavily promoted by health systems and becoming the standard of care, and insofar as patients who access them appear more likely to achieve better outcomes, the research products of ECLIPPSE will undoubtedly support health systems' journeys to reduce HL-related disparities in diabetes care.
